# *ESR1* Variants and Subcontinental Genomic Ancestry: Insights from the 1000 Genomes Project and Native American Populations

**DOI:** 10.1002/cpt.3681

**Published:** 2025-04-17

**Authors:** Mariana M. Scudeler, Caíque Manóchio, Bruno Miwa, Guilherme Belfort‐Almeida, Lucas Faria‐Costa, Cesar Sanchez, Carlos Padilla, Omar Caceres, Eduardo Tarazona‐Santos, Heinner Guio, Timothy D. O'Connor, Fernanda Rodrigues‐Soares

**Affiliations:** ^1^ Pathology, Genetics and Evolution Department, Biological and Natural Sciences Institute Federal University of Triângulo Mineiro Uberaba Brazil; ^2^ Genetics, Ecology and Evolution Department, Biological Sciences Institute Federal University of Minas Gerais Belo Horizonte Brazil; ^3^ Laboratório de Innovación y Desarrollo Instituto Nacional de Salud Lima Peru; ^4^ Universidad de Huanuco Huanuco Peru; ^5^ Institute for Genomes Sciences University of Maryland School of Medicine Baltimore Maryland USA; ^6^ Department of Medicine University of Maryland School of Medicine Baltimore Maryland USA; ^7^ Program in Personalized Genomic Medicine University of Maryland School of Medicine Baltimore Maryland USA

## Abstract

The *ESR1* gene is relevant in breast cancer treatments in the pharmacogenetics context. However, Native, African, and mixed populations are known to be underrepresented in genomic studies. This is particularly important given that the difference in variants' frequencies among different populations can lead to population‐specific clinical implications. Therefore, this study aims to infer the genomic subcontinental ancestry and allele frequencies of the *ESR1* gene variants in 2,427 individuals from 26 populations worldwide from the 1000 Genomes Project and 125 Natives from Peru, whose genomes have not yet been analyzed in the literature regarding this gene. Linear regression with Bonferroni correction analyses was conducted based on ancestry inference and frequencies. Our findings demonstrate subcontinental differentiation of African, Asian, European, and Native populations. Overall, 102 associations (*P* < 0.01) were found for 68 clinically relevant variants. Particularly, subcontinental associations were observed for variants associated with the Native, Asian, European, and African components. We highlight the findings for the rs9349799 and rs2234693 variants, previously associated with altered responses to breast cancer treatments. rs9349799 was positively associated with the South‐Asian component, while rs2234693 was negatively associated with the Coast/Amazonian Native and positively associated with the East‐African component. Nearly half of the variants are intronic, highlighting the importance of studying whole genomes rather than just exomes. These results emphasize subcontinental differences' relevance for designing pharmacogenetic panels. Including neglected populations in genomic and pharmacogenomic studies is essential for democratic access to scientific advances and for more egalitarian and effective pharmacogenetic implementation, tailored to each population's specificities.

**Study Highlights**

**WHAT IS THE CURRENT KNOWLEDGE ON THE TOPIC?**

European populations are significantly overrepresented in genomic studies, while Native and African populations are largely neglected. Given the heterogeneous variant frequencies across populations, this may lead to the oversight of potential population‐specific clinical implications.

**WHAT QUESTION DID THIS STUDY ADDRESS?**

This study investigated the association between genomic subcontinental ancestry and allele frequencies of *ESR1* variants in 1000 Genomes Project populations and original data from Peruvian Natives, focusing on clinically relevant variants.

**WHAT DOES THIS STUDY ADD TO OUR KNOWLEDGE?**

We observed subcontinental ancestry differentiation in *ESR1* variant distributions. Importantly, we have included original data of the Peruvian Native population as part of the efforts to include neglected populations in genomic studies. The study also shows that nearly half of the clinically relevant variants are intronic, emphasizing the need for whole‐genome analyses. Most of the associations were with African components. Also, rs9349799 was positively associated with the South‐Asian component, while rs2234693 was negatively associated with the Coast/Amazonian Native and positively associated with the East‐African component. Both variants were previously associated with altered responses to breast cancer treatments.

**HOW MIGHT THIS CHANGE CLINICAL PHARMACOLOGY OR TRANSLATIONAL SCIENCE?**

Our findings support population‐specific pharmacogenetic panels to reduce the risks of extrapolating genetic data from one population to another. This reinforces the importance of including diverse populations in genomic research for more equitable and effective clinical applications.

According to the World Health Organization (WHO), breast cancer is the first or second leading cause of death due to cancer among women in 95% of the world's countries. By 2040, it is estimated that 3 million new cases and 1 million deaths will be reported annually worldwide.^1^ Several factors contribute to the development of this disease, including genetics, such as mutations in the *BRCA1* and *BRCA2* genes, and hormonal factors, such as estrogen exposure.^2^, ^3^ This hormone acts mainly by binding to two specific intracellular receptors, ERα and ERβ, predominantly to ERα, stimulating the expression of genes that promote cell proliferation and inhibit apoptosis.^4^, ^5^ These receptors are encoded by the *ESR1* and *ESR2* genes, which, along with *HSD17B1* and *CYP19A1*, play a role in the aromatase inhibitor pathway. This pathway is targeted by a class of drugs designed to reduce endogenous estrogen production, called aromatase inhibitors. The third generation of these drugs includes letrozole, anastrozole, and exemestane. This pathway is also targeted by selective estrogen receptor modulators (SERMs), such as tamoxifen, raloxifene, and toremifene, which act as agonists or antagonists of estrogen receptors.^3^, ^4^, ^6^

However, drug response can vary significantly among patients. For a small subgroup, drugs might be ineffective or lead to adverse effects, including death. This is due to several specific factors that can result in interindividual variability in treatment response, including transient environmental and physiological influences, as well as genetic factors. Genetic variants that lead to changes in enzymes, receptors, and transporters involved in drug pharmacokinetics and pharmacodynamics are possibly the main cause of interindividual variability.^7^

Pharmacogenetics (PGx) aims to understand the association between variants in pharmacogenes, which are genes of pharmacogenetic interest, and variations in treatment response.^8^ The rs9322336 variant of the *ESR1* gene, for instance, was associated with musculoskeletal syndrome and treatment discontinuation in patients treated with exemestane.^9^ Musculoskeletal adverse effects were also associated with rs2234693 and rs9340799 in response to the use of letrozole and anastrozole in a group of East‐Asian patients.^10^ The development of arthralgia associated with the three aforementioned variants following the prescription of letrozole and anastrozole has also been reported.^11^ Ethnicity is another influencing factor regarding interindividual variability in drug response, as variant frequencies in pharmacogenes are heterogeneous across different populations, potentially leading to specific clinical implications.^8^, ^12^, ^13^, ^14^ However, it is important to emphasize that an individual's self‐reported ethnicity may not necessarily correspond to their genomic ancestry, that is, their population genetic origins.^15^, ^16^ This is particularly relevant for mixed populations, such as in Brazil,^16^ which, similar to Latin America in general, has ethnic roots derived from three main parental populations: Africans, Europeans, and Native Americans.^8^, ^17^

Regarding specific clinical implications, one of the prominent examples is warfarin, an anticoagulant widely used in clinical practice. It is known that the required dosage for therapeutic effect varies among African‐Americans, Europeans, and Asians due to frequency variations in variants of the two major genes involved in its response ‐ *CYP2C9* and *VKORC1*.^8^, ^18^ Recently, Moreira *et al*.^19^ discussed that an extra dose of chemotherapy may be necessary for patients of Native‐American ancestry with acute lymphoblastic leukemia to achieve the expected efficacy. Despite this, it is widely acknowledged that non‐European populations, such as Native Americans, are underrepresented in genetic and genomic studies, with predominantly European overrepresentation.^8^, ^19^, ^20^, ^21^ Including underrepresented populations in studies is crucial for designing panels that address the specific characteristics of each population, as selecting an extremely rare variant in a given population would represent an inefficient and unjustified allocation of resources. Therefore, considering the interpopulational differences in pharmacogene polymorphism frequencies and the potential of *ESR1* genotyping for the efficacy and safety of hormonal treatments for breast cancer patients, we aimed to investigate the association between the frequency of *ESR1* gene variants and the subcontinental ancestry of different world populations, including Native populations not yet studied in this context to the best of our knowledge. This approach could, in the future, make the clinical implementation of pharmacogenetics more effective, precise, cost‐affordable, and truly equitable for diverse populations around the world.

## MATERIALS AND METHODS

The phase III 1000 Genomes public database^22^ sourced the genomes of 2,427 unrelated individuals from 26 different populations worldwide. These include: 91 British individuals living in England and Scotland (GBR), 99 Finns (FIN), 51 individuals of European descent living in Utah (CEU), 107 individuals from Tuscany, Italy (TSI), 107 Iberians from Spain (IBS), 104 Japanese from Tokyo (JPT), 107 Han Chinese from the southern region (CHS), 103 Han Chinese from Beijing (CHB), 91 Dai Chinese from Xishuangbanna, China (CDX), 99 Kinh Vietnamese (KHV), 102 Gujarati Indians in Houston, TX (GIH), 102 Telugu Indians living in England (ITU), 102 Sri Lankan Tamil individuals living in England (STU), 86 Bengali individuals in Bangladesh (BEB), 96 Punjabis from Lahore, Pakistan (PJL), 112 Mandinka from Gambia (GWD), 78 Mande individuals from Sierra Leone (MSL), 108 Yoruba from Nigeria (YRI), 95 Esan from Nigeria (ESN), 88 Luhya individuals from Kenya (LWK), 96 Afro‐Caribbeans from Barbados (ACB), 56 African‐Americans from the southwestern United States (ASW), 85 Peruvians from Lima, Peru (PEL), 64 individuals of Mexican descent from Los Angeles (MXL), 94 Colombians from Medellín (CLM), and 104 Puerto Ricans (PUR). Furthermore, we included original whole‐genome sequencing data from 125 unrelated Native‐American individuals from the Matzes, Moches, Trujillo, Iquitos, Cusco, Chopccas, and Uros populations (*n* = 9; 25; 15; 16; 16; 25, and 19, respectively).^23^ Both datasets were aligned to the GRCh37.p13 version.

The 1000 Genomes Project data were downloaded to the Laboratory of Human Genetic Diversity (LDGH) server at the Federal University of Minas Gerais (UFMG). Both datasets were filtered for quality control to remove A|T and C|G variants, heterozygous variants, duplicates, and any missing data. This process was carried out using the MosaiQC script (https://github.com/ldgh/MosaiQC‐public).

To perform Admixture analysis, both datasets were merged and filtered^24^ to exclude one variant per pair in linkage disequilibrium (LD) (value *r* ^2^ ≥ 0.4) and with a minor allele frequency (MAF) < 0.05 using Plink 1.9 software.^24^ Additional filters were applied to exclude duplicates, non‐biallelic variants, and those with missing data, resulting in 525,210 variants per individual. The KING software^25^ was used to estimate the kinship coefficient between the Peruvian Native individuals, while NAToRA^26^ was employed to exclude those with a kinship degree equal to or > 0.0625. The Admixture software^27^ was used in the unsupervised mode for ancestry inference, setting the number of ancestry components to range from 3 to 10 (*k* = 3–10). Cross‐validation analyses were conducted to determine the optimal number of components, identified by the lowest cross‐validation value, which was *k* = 9 (cv = 0.47759). The software generated an output with the percentages of each ancestry component for each individual. Using this output, the average ancestry of each population was calculated with the mean function on the R platform (www.r‐project.org) .^23^

The *ESR1* gene was filtered using the *vcftools* tool^28^ based on the position described by the National Center for Biotechnology Information (NCBI) (151977807…152450754). The resulting VCF files contained 13,752 variants (1,000 Genomes) and 2,281 variants (original Native‐American data). Allele frequencies were calculated on the R platform using the *makefreq* function from the adegenet package.^29^ Linear regression with Bonferroni correction (adjusted *P*‐value = 0.01) between allele frequencies and subcontinental ancestry (*k* = 9) was conducted using the *lm()* function on the R platform. The scripts used are available at https://github.com/marianascudeler. These analyses were based on the methodology of Rodrigues‐Soares *et al*.^30^

**Figure 1:**
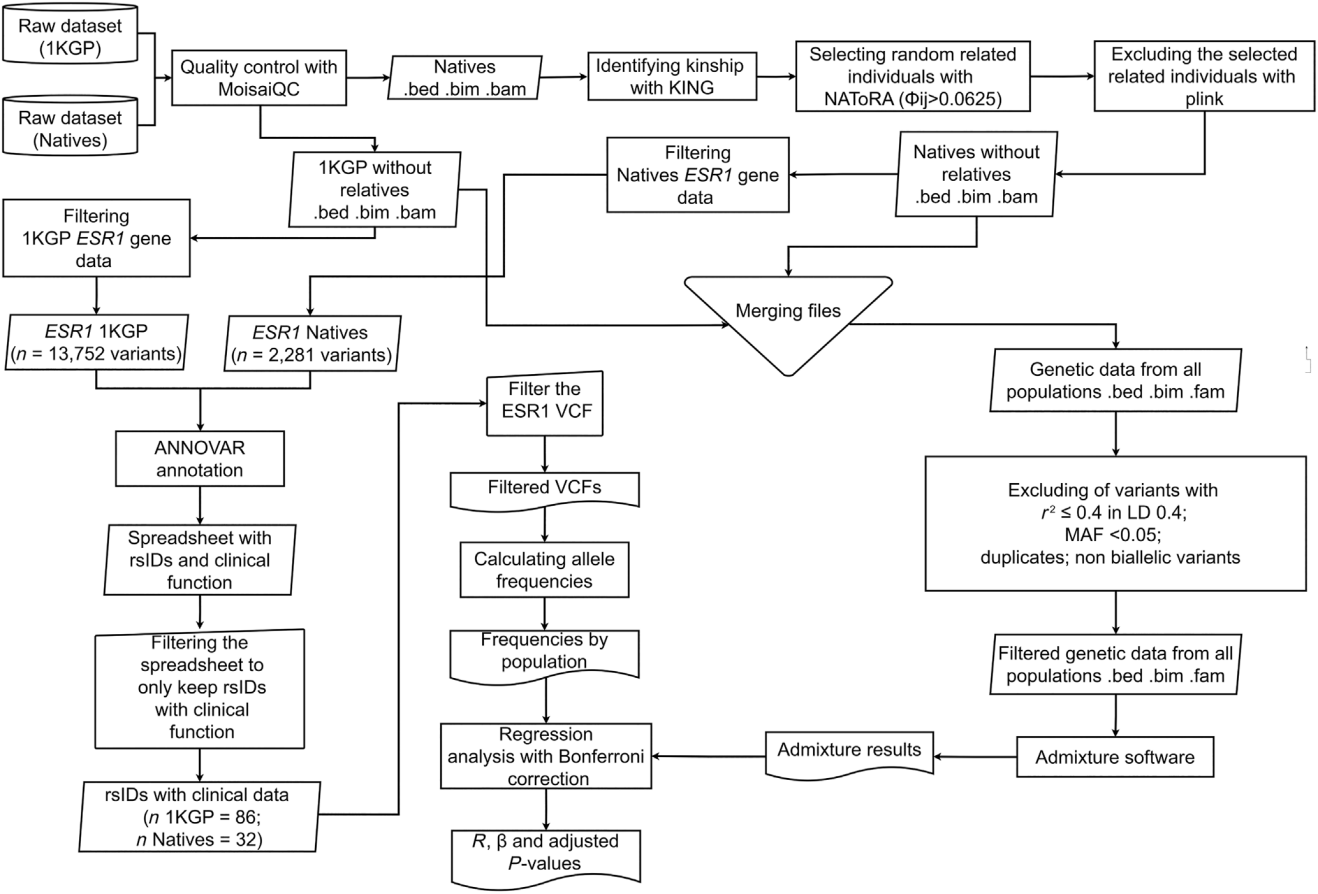
Flowchart showing the steps involved in the study methodology. 1KGP, 1000 Genomes Project; LD, linkage disequilibrium; MAF, minor allele frequency.

The variants were annotated using the Annovar software,^31^ utilizing the refGene 2020‐08‐17,^32^ clinvar_20221231,^33^ dbsnp150,^34^ Combined Annotation Dependent Depletion (CADD) 1.7, and AlphaMissense from dbNSFP 4.7a^35^ databases. Variants with known associations with clinical data from ClinVar^33^ and PharmGKB^36^ were discussed and analyzed. The flowchart showing the steps involved in the analysis is shown in **Figure** **1**.

## RESULTS AND DISCUSSION

### Ancestry

**Figure 2:**
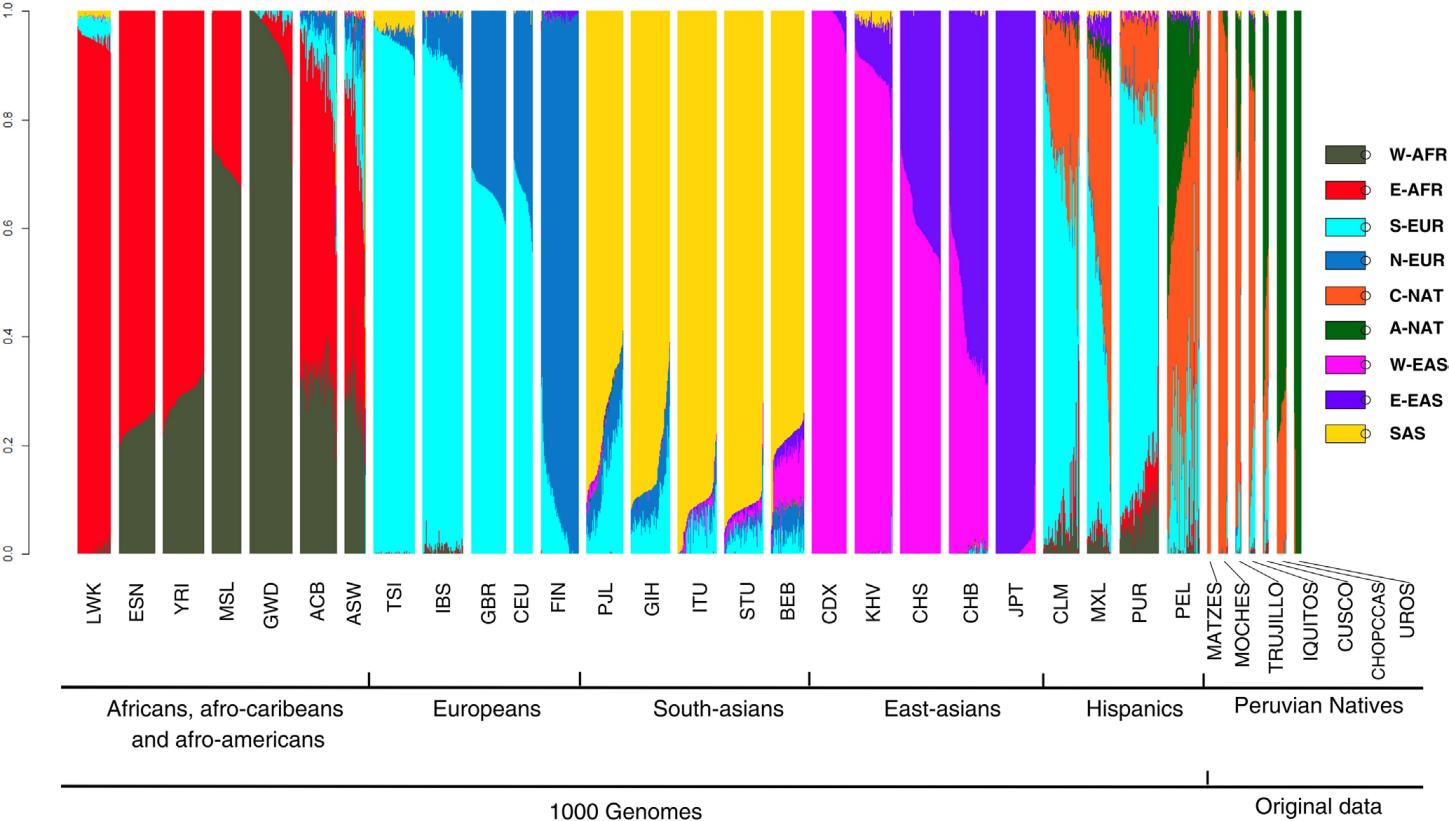
Ancestry bar graph by population. ACB, Afro‐Caribbeans from Barbados; A‐NAT, Andean Native‐American2; ASW, Individuals with African ancestry in the southeastern US; BEB, Bengalis from Bangladesh; CDX, Dai Chinese from Xishuangbanna; CEU, European descendants from Utah; CHB, Han Chinese from Beijing; CHS, Han Chinese from the South; CLM, Colombians from Medellín in Colombia; C‐NAT, Coast/Amazonian; E‐AFR, East‐African; E‐EAS, East‐Asian; ESN, Esan from Nigeria; FIN, Finns from Finland; GBR, Britons from England and Scotland; GIH, Gujarati Indians in Houston, USA; GWD, Mandika from Gambia; IBS, Iberians from Spain; ITU, Telugu Indians in the UK; JPT, Japanese from Tokyo; KHV, Kinh Vietnamese from Ho Chi Minh City; LWK, Luhya from Kenya; MSL, Mende from Sierra Leone; MXL, Individuals with Mexican ancestry living in Los Angeles; N‐EUR, North‐European; PEL, Peruvians from Lima; PJL, Pakistanis; PUR, Puerto Ricans from Puerto Rico; SAS, South‐Asian; S‐EUR, South‐European; STU, Sri Lankan Tamils in the UK; TSI, Italians from Tuscany; W‐AFR, West‐African; W‐EAS, West‐Asian; YRI, Yoruba from Nigeria.

Considering the ancestry inference with *k* = 9 performed for the 2,427 individuals from 26 populations worldwide from the 1000 Genomes Project and the 125 individuals from the 7 Native‐American populations, we observed subcontinental differentiation among the defined components (**Supplementary Material** **1**). Specifically, subcontinental differentiation was evident in the establishment of three Asian components [West of East Asia (W‐EAS), East of East Asia (E‐EAS), and South‐Asian (SAS)], two African components [West‐African (W‐AFR) and East‐African (E‐AFR)], two European components [North‐European (N‐EUR) and South‐European (S‐EUR)], and two Native‐American components [Coast/Amazonian (C‐NAT) and Andean (A‐NAT)] (**Figure** **2**). Similar differences have been observed in multiple studies throughout the literature, particularly in European, African, and Asian populations.^22^, ^37^, ^38^, ^39^, ^40^, ^41^, ^42^

**Figure** **2** shows the African populations with two subcontinental components: W‐AFR and E‐AFR. There is an apparent and gradual decrease in the proportion of the E‐AFR component from east to west across the continent, seen in the populations of Kenya (94.4%), Nigeria (76.41%—ESN; 70.98%—YRI), Sierra Leone (28.91%), and Gambia (4%). Auton *et al*.^22^ performed a similar analysis using the same populations from the 1000 Genomes Project, defining *k* values between 5 and 12 using a set of fewer SNPs. Their results for *k* = 9 are similar. Notably, starting from *k* = 10, the African populations are differentiated into three subcontinental components, with the populations of Kenya (LWK), Nigeria (ESN and YRI), and Gambia (GWD) being well separated, each with its own component.^22^ Gouveia *et al*.^43^ analyzed 6,267 individuals with more than 10% African ancestry from 25 populations: 9 mixed populations from the Americas, 11 African populations, 2 European populations, and 3 Native‐American populations. The data came from the EPIGEN‐Brazil project, the 1000 Genomes Project, Prostate, Lung, Colorectal, and Ovarian Cancer Screening, the Ghana Prostate Study Accra, Epidemiology of Burkitt's Lymphoma, the Peruvian National Institute of Health, and the Tishkoff Laboratory. The authors established four African components: west‐central, south/eastern, western, and northern Uganda.^43^ Unlike our analysis and that of Gouveia *et al*. and Kehdy *et al*., some studies have not observed the mixture of two components in the Yoruba population.^38^, ^39^, ^40^, ^41^, ^42^, ^44^ This discrepancy may be due to differences in the set of SNPs, higher *k* values, or the number of studied individuals and populations. Additionally, some authors have not observed the separation between the Luhya and Yoruba populations found in our analyses.^39^, ^40^, ^41^, ^43^

The mixed populations of African origin [Afro‐Caribbeans (ACB) and African‐Americans from the United States (ASW)] present similar proportions of both African components. In their analysis, Gouveia *et al*.^43^ also observed very close proportions of the ancestral components of ACB and ASW, except for a third African component (southern/eastern).

Regarding European populations, the observed subcontinental separation between north and south aligns with previous literature, reinforcing the importance of considering European populations as mixed at a subcontinental level to avoid potential spurious findings in genetic studies.^22^, ^37^, ^38^ Similar to African populations, there is a clear and gradual decrease in the proportion of the S‐EUR component as one moves from south to north across the continent (94.28% in Italians, 89.98% in Spaniards, 66.55% in English and Scots, and 8% in Finns). This pattern has also been observed by Homburger *et al*.^38^ Another study assessing subcontinental European ancestry identified three components: North‐European, South‐East‐European, and South‐West‐European. The authors noted a marked separation of the Finnish population, consistent with our findings.^37^ Among the CEU population, composed of individuals of European descent living in Utah, the proportions are very similar to those seen in the English and Scots (66.55% vs. 65.95% for S‐EUR and 33.42% vs. 33.93% for N‐EUR, respectively), reflecting the formative history of the US population, which was colonized by English people.^45^ This observation is also supported by Gouveia *et al*. and Auton *et al*.^22^, ^37^

Within the Asian populations, subcontinental differences separated the components into three groups: one South Asian and two East Asians. The South‐Asian populations were well differentiated from the East‐Asian populations, aligning with observations by Pagani *et al*.^41^ Among the East‐Asian populations, the Japanese differed significantly from the Chinese (with 98.78% of the E‐EAS component for the Japanese population). Among the Chinese populations, differentiation was evident between ethnic groups, with 1%, 38.19%, and 56.64% of the E‐EAS component for the Southern Dai, Southern Han, and Beijing Han populations, respectively. This differentiation has divided East‐Asian components into East and West. Lazaridis *et al*. and Tishkoff *et al*. did not observe such considerable differences between Chinese and Japanese groups.^40^, ^44^ Conversely, a study assessing the genetic structure and ancestry of Chinese, Korean, and Japanese populations concluded that these populations could be well distinguished using both the whole‐genome approach and ancestry informative markers (AIMs). Differences between the Dai and Han ethnic groups were also established, consistent with our analyses.^46^

Concluding the 1000 Genomes Project populations, Hispanics include Colombians (CLM), Mexicans living in the United States (MXL), Puerto Ricans (PUR), and Peruvians (PEL). For all these populations, a greater number of components (i.e., ancestries) can be observed when compared to the other populations, which is coherent with the fact that these are mixed populations of similar ancestral origins (Native American, European, and African).^38^ Notably, the high proportion of native ancestry in the Peruvian population stands out. A previous study evaluating the demographic history and ancestry of South America reported that the Peruvian population has the highest proportion of Native‐American ancestry among South American populations, specifically from the Andes.^38^ However, our analysis resulted in a lower proportion (27.03%) of Andean ancestry (A‐NAT) in the Peruvians from the 1000 Genomes Project. The Lima population analyzed by Harris *et al*.^23^ showed a similar proportion of native ancestry with the presence of a European component.

Considering the seven populations derived from original data from Peruvian Natives (Matzes, Moches, Chopccas, Uros, Trujillo, Iquitos, and Cusco), we observed subcontinental separation within these populations based on the Native components (C‐NAT and A‐NAT). The Moches population from the coastal region of Peru proved to be very different from the Uros in the Andes. The proportion of the C‐NAT component, defining the Moches, decreases progressively in relation to the A‐NAT component as one moves from the coast, through the Amazon region, and into the Andes. In 2018, Harris *et al*. published the most extensive panel of whole‐genome sequencing data from Native populations, which included the 125 genomes analyzed here. They also used genotyping array data from over 130 individuals with high Native‐American ancestry to evaluate evolutionary genomic dynamics. Ancestry analyses conducted by Harris *et al*. ranged from *k* = 4 to 8, with results at *k* = 7 closely resembling those found in our study, particularly regarding the differentiation between the Moches and Uros populations. However, this similarity was not observed at other *k* values (4, 5, 6, and 8), likely due to differences in the selected SNP sets: our study used 525,210 variants, whereas Harris *et al*.^23^ used 183,579 variants. Homburger *et al*.^38^ also demonstrated clear differentiation between Native Andean and non‐Andean populations utilizing a set of 364,470 SNPs.

### *ESR1* variants annotation

The 1000 Genomes Project and Native VCF *ESR1* files contained 13,752 and 2,281 variants, respectively. After annotating the data using the ANNOVAR software,^47^ variants were filtered based on the availability of clinical data in ClinVar and the Clinical Annotations tab of PharmGKB.^33^, ^36^ This resulted in the selection of 86 variants from the 1000 Genomes Project and 32 from the Native data. The frequencies of these selected variants were calculated, and regression and correlation analyses were conducted between their frequencies and the nine ancestry components.

Of the 13,752 variants identified from the 1000 Genomes Project data, 12,345 were located in intronic regions, 62 in exonic regions, 260 in the 3′ UTR, and 33 in the 5′ UTR. Among the 2,281 variants derived from the original Native Peruvian data, 2,028 were located in intronic regions, 12 in exonic regions, 40 in the 3′ UTR, and 6 in the 5′ UTR. Regarding the selected variants, the annotation analysis revealed that about half of the analyzed variants (*n* = 42–48.8% from the 1000 Genomes Project and 16–50% from the Peruvian Native data) are located in intronic regions, contrasting with 32.5% and 28.1% of variants in exons (28 and 9 variants, respectively). It is worth highlighting the work by Yang *et al*.,^48^ where the authors aggregated data on annotated pharmacogenetic variants.^36^, ^49^, ^50^ Their analysis found that 48% (*n* = 1,566) of the pharmacogenetic variants were in intronic regions, reinforcing the importance of studying complete genomes rather than just exomes, as the results found here. Given the proportion of intronic and exonic variants observed among the total 13,752 and 2,281 variants, both subsets of variants with clinical annotations can be considered enriched for exonic variants.

Regarding the refGene database,^32^ out of the 86 included variants from the 1000 Genomes Project, 66 were listed as possibly associated with estrogen resistance and 19 with Emery‐Dreifuss muscular dystrophy and spinocerebellar ataxia. As for the variants in the Native data, 24 of 32 variants were listed as possibly associated with estrogen resistance and 8 with Emery‐Dreifuss muscular dystrophy and spinocerebellar ataxia.

Additionally, we incorporated data from the CADD database.^35^ The results are expressed through the PHRED‐scaled C‐score, a logarithmic value used to identify potentially pathogenic variants. In agreement with the original authors, we established a cutoff of > 20, which indicates that the variant is among the top 1% of most deleterious mutations in the genome. The higher the PHRED score, the greater the probability that the variant has a functional impact and is potentially pathogenic. Considering the variants from the 1000 Genomes Project, rs139960913, rs200075329, rs149308960, rs77797873, rs139740651, rs2295190, rs144206837, and rs142712646 were selected based on the defined cutoff. Notably, rs142712646, with a PHRED score of 32, falls within the top 0.1% of most deleterious variants, indicating over 90% probability of pathogenicity. However, the CADD authors recommend that these results should be assessed in conjunction with additional evidence. Therefore, we also annotated the variants using the AlphaMissense database,^35^ which likewise classified rs142712646 as likely pathogenic, along with rs139960913. In contrast to the CADD annotation, rs2295191, rs144206837, rs2295190, rs139740651, rs77797873, rs149308960, and rs200075329 were classified by AlphaMissense as likely benign, along with rs9340773, rs201145204, rs185717042, rs139548761, rs2295192, and rs572679653. Finally, regarding the variants identified in the original Peruvian Native dataset, rs200075329, rs17847065, rs2295190, and rs747405346 presented PHRED scores > 20. Among these, rs747405346 was also classified by AlphaMissense as likely pathogenic, while rs200075329, rs17847065, and rs2295190, along with rs778554662 and rs2295192, were classified as likely benign.

Nevertheless, it is important to note an annotation limitation that should be considered in future studies and when designing annotation analyses. According to the NCBI for reference genome 37, there is a particular region of overlap between the *ESR1* and *SYNE1* genes on opposite strands. Given that a polymorphism always occurs at the same locus on both strands of DNA, an *ESR1* polymorphism can be identified as a *SYNE1* polymorphism if the analysis does not specify which strand is being analyzed, and vice versa. Consequently, we observed that from the 86 and 32 variants filtered for our analysis, 19 and 8 were annotated as variants of the *SYNE1* gene, respectively. To improve future annotation processes, the limitation of gene overlaps should be considered for maximum annotation accuracy.

### Association between ancestry and allele frequencies and annotated data

**Figure 3:**
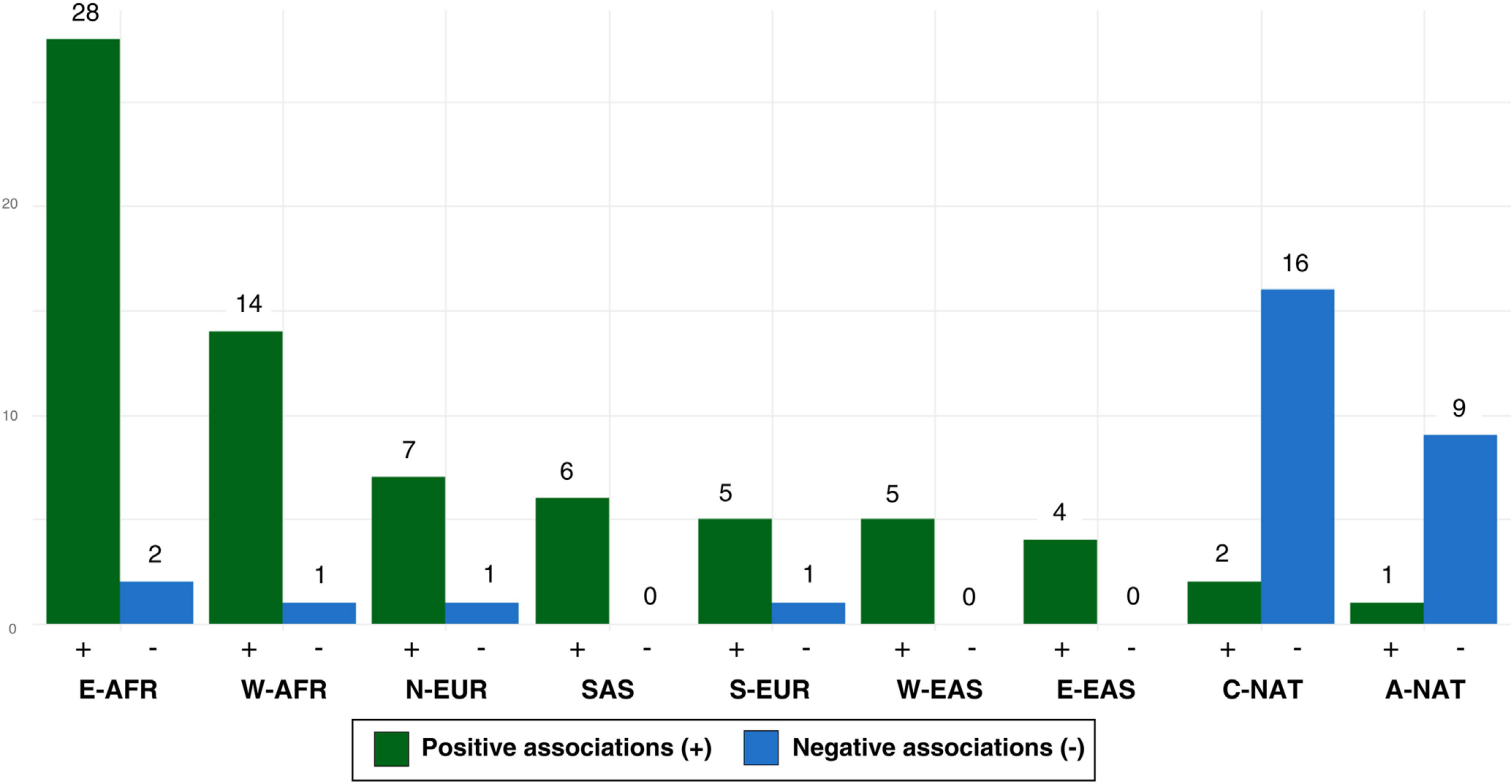
Number of positive and negative associations by ancestry component. A‐NAT, Andean Native‐american; C‐NAT, Coast/Amazonian Native‐american; E‐AFR, East‐African; E‐EAS, East‐Asian‐East; N‐EUR, North‐European; SAS: South‐Asian; S‐EUR, South‐European; W‐AFR, West‐African; W‐EAS, East‐Asian‐West.

Based on the linear regression and Pearson correlation analysis performed between the allele frequencies of *ESR1* variants and the nine ancestry components (W‐AFR, E‐AFR, S‐EUR, N‐EUR, W‐EAS, E‐EAS, SAS, C‐NAT, and A‐NAT), we observed a total of 102 significant associations (*P* < 0.01) for 68 variants (**Supplementary Material** **1**). Of these associations, 72 are positive (for 57 variants), predicting that the higher the proportion of a given ancestry, the greater the chances of the individual carrying these variants. Conversely, 30 associations are negative (for 19 variants), indicating the opposite: the higher the proportion of ancestry, the lower the chances of carrying the variant (70.5% and 29.4% of the associations, respectively, **Figure** **3**).

As for the associations with the nine components, 45% were found in the African ancestry components (W‐AFR and E‐AFR) (**Figure** **3**). Given the substantial number of associations and the role of *ESR1* in pathways of considerable relevance within the hormonal treatment of breast cancer, one striking point is that women with African ancestry have a higher risk of mortality from the disease. Considering the underrepresentation of African populations in genomic and breast cancer pharmacogenetic studies, this reinforces the call for the inclusion of such populations in genetic studies, as this may enable finding ways of filling gaps on this issue within genetics and pharmacogenetics.^5^, ^51^

To more deeply evaluate the extent of the subcontinental differences in the associations between the allele frequencies of *ESR1* variants and the genomic ancestry components, we observed that, when considering the variants by continental cluster (i.e., evaluating SAS + W‐EAS + E‐EAS, W‐AFR + E‐AFR, N‐EUR + S‐EUR, and C‐NAT + A‐NAT), some variants were exclusive to the subcontinental component they were associated with, compared to its other continental components (**Supplementary Material** **2**). Importantly, “exclusive” implies that the variant was associated solely with one component of the continental cluster. In other words, variants whose frequencies were associated with, for example, the subcontinental component SAS and not with W‐EAS and E‐EAS or other clusters (**Table** **1**). Conversely, “non‐exclusive” indicates that the variant has been associated with all the components of the continental cluster. These findings underscore the importance of subcontinental differences in the context of pharmacogenetic implementation, including the design of pharmacogenetic panels, while also highlighting the dangers of extrapolating genetic data from one population to another.^44^, ^51^, ^52^, ^53^, ^54^ In contrast, the opposite trend is seen when considering the African components: a higher number of variants are associated with both African components (i.e., non‐exclusive), indicating greater continental similarity.

**Table 1:** Number of exclusive and non‐exclusive variants per ancestry component among the continental clusters (SAS + W‐EAS + E‐EAS; C‐NAT + A‐NAT; S‐EUR + N‐EUR; W‐AFR + E‐AFR)

	Number of associated variants
Asian components	
SAS exclusives	4
W‐EAS exclusives	2
E‐EAS exclusives	1
Non‐exclusives	5
Native components	
A‐NAT exclusives	1
C‐NAT exclusives	4
Non‐exclusives	14
European components	
S‐EUR exclusives	2
N‐EUR exclusives	5
Non‐exclusives	4
African components	
W‐AFR exclusives	4
E‐AFR exclusives	14
Non‐exclusives	16

A‐NAT, Andean Native‐american 2; C‐NAT, Coast/Amazonian Native‐american; E‐AFR, East‐African; E‐EAS, East‐Asian ‐ East; N‐EUR, North‐European; SAS, South‐Asian; S‐EUR, South‐European; W‐AFR, West‐African; W‐EAS, East‐Asian – West.

### Variants listed on ClinVar and PharGKB

Regarding variants annotated with clinical data available in ClinVar^33^ by multiple submissions, we highlight that rs12681 (positively associated with E‐AFR–**Supplementary Material** **2**), rs2250122 (negatively associated with W‐AFR and E‐AFR), rs200880341 (positively associated with E‐EAS), rs2295190 (positively associated with N‐EUR), rs2295191 (positively associated with E‐EAS and W‐EAS) and rs2295192 were listed as benign or probably benign for autosomal recessive ataxia Beauce type and Emery‐Dreifuss muscular dystrophy. Regarding the same conditions, rs567194577 and rs144206837, in turn, were considered variants with conflicting pathogenicity data. The rs2077647 and rs2228480 variants (positively associated with C‐NAT) have been listed as benign or probably benign for susceptibility to migraine with or without aura, myocardial infarction, estrogen resistance syndrome, and hereditary breast cancer. Furthermore, rs200075329, rs78574022 (positively associated with E‐AFR), and rs9341068 (positively associated with W‐AFR and E‐AFR) were considered benign or probably benign. Meanwhile, rs139740651 (positively associated with E‐AFR) and rs139960913 (positively associated with N‐EUR) have uncertain significance and conflicting pathogenicity data, respectively. As for the variants annotated in the *Clinical Annotations* tab of PharmGKB,^6^ we point out the rs2234693 variant that, in addition to being classified as a risk factor for myocardial infarction by ClinVar,^33^ is associated with the risk of toxicity due to the use of letrozole and anastrozole for the treatment of neoplasms and methamphetamine for the treatment of psychotic illnesses, along with an unsatisfactory response to the treatment of rheumatoid arthritis with leflunomide (CC genotype). The rs9340799 variant, on the other hand, is associated with toxicity risk following the use of tamoxifen, letrozole, and anastrozole, and a decreased response to leflunomide and greater increase in spinal bone mineral density in response to hormone replacement with conjugated estrogen and medroxyprogesterone (GG genotype). The rs2207396 variant is associated with toxicity due to the treatment of neoplasms with alkylating agents and cisplatin, which is negatively associated with C‐NAT and A‐NAT and positively associated with SAS. Similarly, rs2813543, negatively associated with both the Native components, is listed as associated with toxicity in response to breast cancer treatment with exemestane, as is rs9322335 regarding exemestane and letrozole and rs4870061 (negatively associated with both Native components) regarding letrozole.

**Figure 4:**
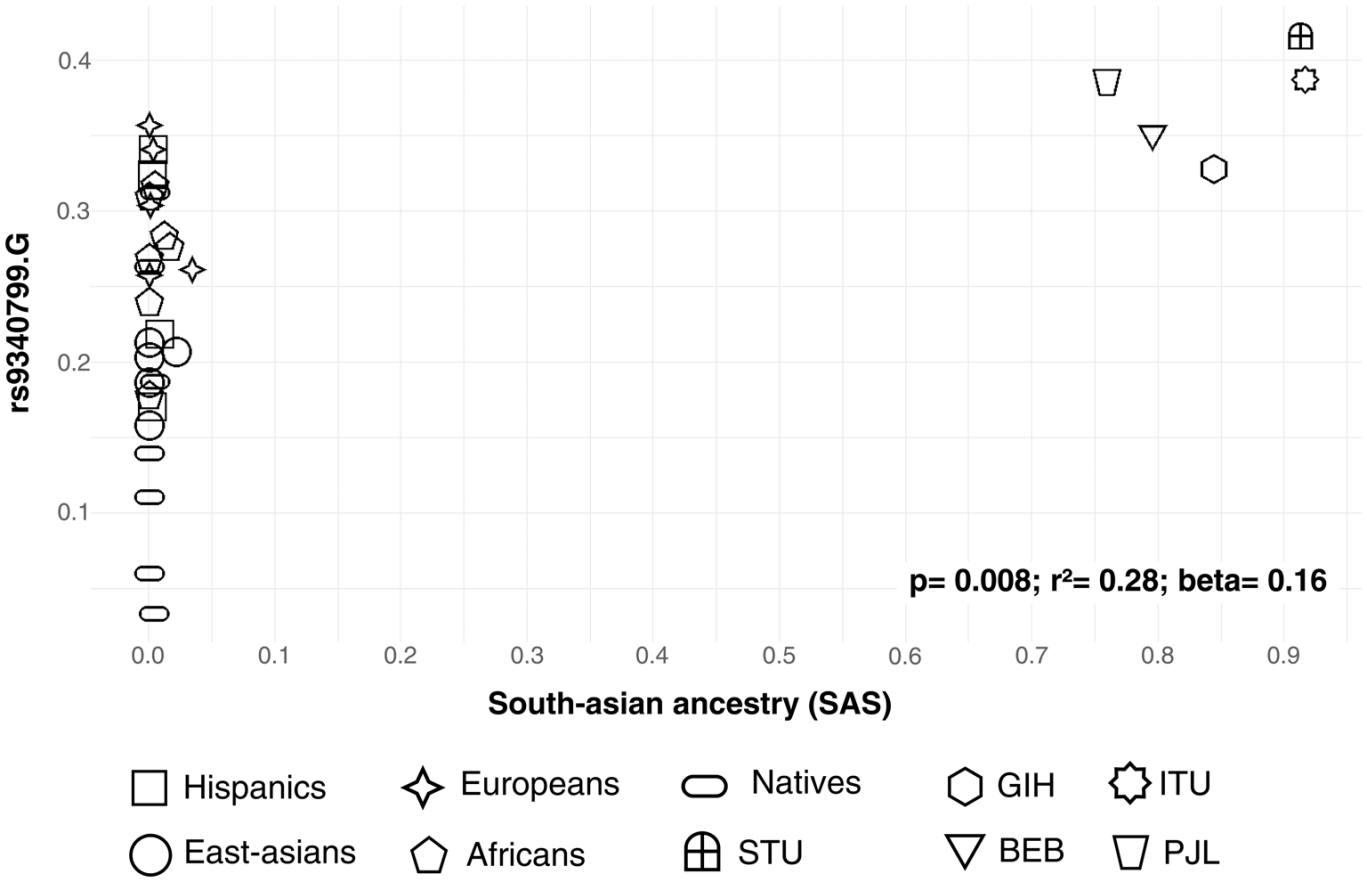
Plot of allele frequency distribution (rs9340799.G) in relation to ancestry: South‐Asian ancestry (SAS). BEB, Bangladeshi Bengalis; GIH, Gujarati Indians in Houston, USA; ITU, Telugu Indians in the UK; PJL, Pakistanis; STU, Sri Lankan Tamils in the UK.

Moreover, we highlight the rs9340799 and rs2234693 variants, which, in addition to the aforementioned findings, were considered the main *ESR1* gene variants associated with altered response to breast cancer treatments in a systematic review by our group focused on pharmacogenetics published in 2022.^5^ According to our regression analyses, rs9340799 is positively associated with the SAS component (*P* = 0.008; *r* ^2^ = 0.28; *β* = 0.1675; **Figure** **4**).

**Figure 5:**
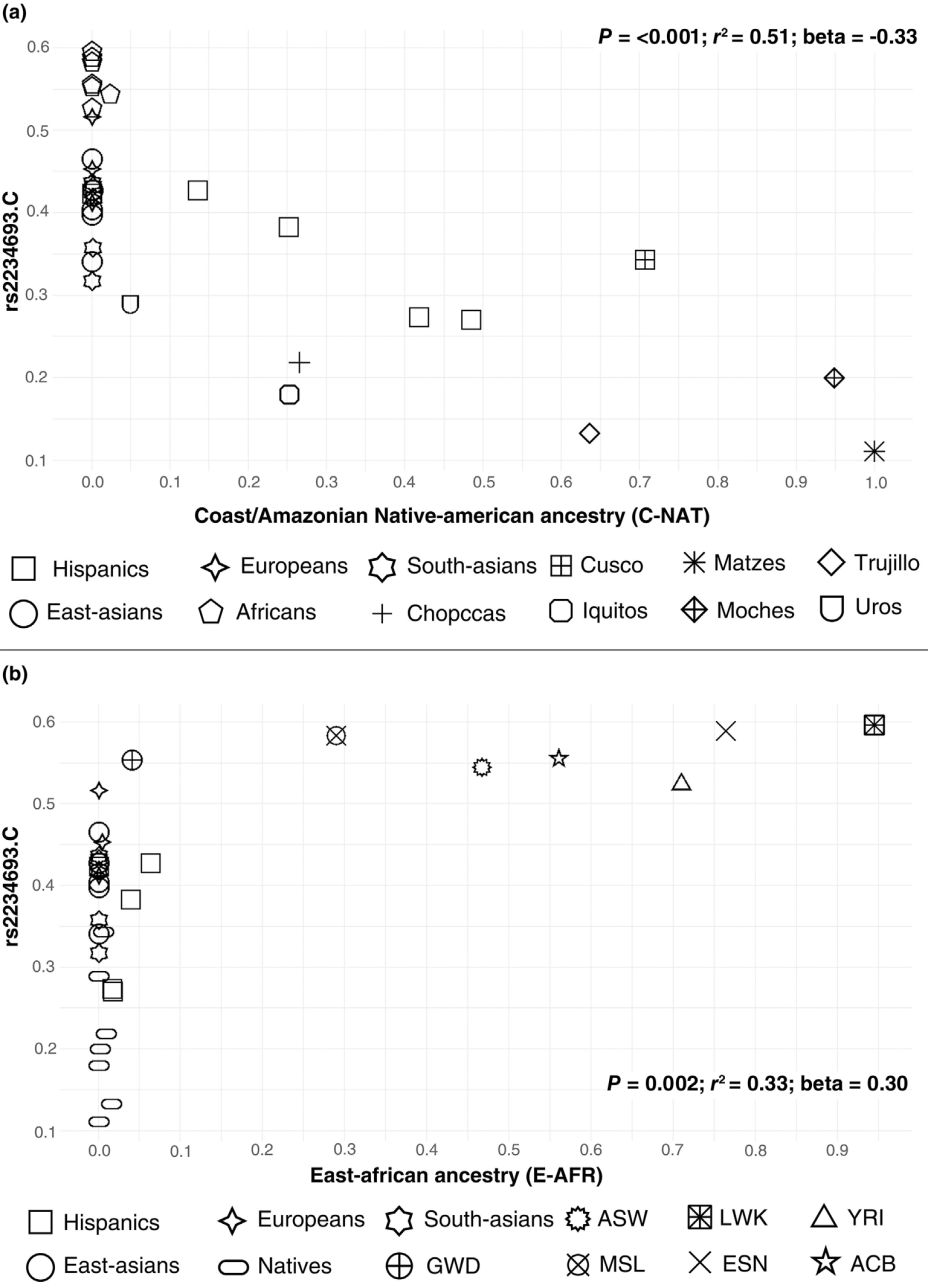
Plot of allele frequency distribution (rs2234693.C) in relation to ancestry: (**a**) Coast/Amazonian Native‐American ancestry (C‐NAT); (**b**) East‐African ancestry (E‐AFR); ACB, Afro‐Caribbeans from Barbados; ASW, individuals with African ancestry in the southeastern USA; ESN, Esan from Nigeria; GWD, Mandika from Gambia; LWK, Luhya from Kenya; MSL, Mende from Sierra Leone; YRI, Yoruba from Nigeria.

The rs2234693 variant, in turn, is also negatively associated with the C‐NAT component (*P* = < 0.001; *r* ^2^ = 0.51; *β* = −0.3358, **Figure** **5** **a**), while it is positively associated with the E‐AFR (*P* = 0.002; *r* ^*2*^ = 0.33; *β* = 0.3049). Here, the African populations stand out from the others (**Figure** **5** **b**).

Native American, African, South Asian, and mixed populations are notably underrepresented in genomic and, specifically, breast cancer pharmacogenetic studies.^5^, ^51^ Future studies must evaluate the role of these variants in clinical and therapeutic contexts, particularly with greater ethnic and population diversity.

Furthermore, we emphasize that the findings found here may be of great use in clinical practice. Ideally, it is hoped to have patient‐specific genetic data available in the context of pharmacogenetic implementation and personalized medicine; however, in the absence of this data, ancestry information can be useful to guide clinical decisions on the patient and their treatment, since linear regression analyses allow the prediction of an individual's chances of carrying certain variants according to their ancestry analysis. This is a fact already established by Ramos *et al*.^12^ in a study in which the authors included 1,478 individuals from 19 populations from different parts of the world (1,000 Genomes and original data from additional African populations) and analyzed 212 genes involved in the absorption, distribution, metabolism, and excretion (ADME) of drugs. Results such as these can be used to mitigate the currently unequal access to pharmacogenetic and genomic scientific advances for non‐European populations while working to ensure that they are adequately represented in the studies.

## CONCLUSION

Our analysis revealed significant subcontinental differences in genomic ancestry among 2,654 individuals from 26 populations in the 1000 Genomes Project and 7 Peruvian Native populations, marking the first comprehensive study of *ESR1* gene sequencing in Native populations. Notably, African, European, Asian, and Native populations were separated subcontinentally. Altogether, 102 associations were found between the variants' frequencies and the nine ancestry components, of which 102 were positive, and almost 50% were associations with the African components (W‐AFR and E‐AFR). Our results resonate with recent findings that European populations should be considered mixed to avoid erroneous interpretations in genetic studies. Furthermore, we highlight that the specificity of associations to subcontinental components underscores and underlines the importance of considering subcontinental differences in the design of pharmacogenetic panels and their clinical implementation, as well as indicating possible clinical implications specific to different ethnicities. From a clinical practice perspective, we emphasize that association results between clinically relevant *ESR1* variants and subcontinental ancestry components can be used to guide clinical decisions on a patient in the absence of their specific genetic data. Nevertheless, our data reinforce the dangers of extrapolating data interpopulationally, especially echoing the efforts to include neglected populations in genomic and pharmacogenomic studies so that pharmacogenetic implementation may, in the future, be more assertive, democratic, and maximally effective.

## FUNDING

This study was supported by the Brazilian Conselho Nacional de Desenvolvimento Científico e Tecnológico (CNPq 440238/2022‐6, 402533/2022‐4, 314344/2020‐9, 407046/2023‐2, 406913/2022‐6, and 312807/2022‐8, (440066/2020–4)), Coordenação de Aperfeiçoamento de Pessoal de Nível Superior (CAPES), Fundação de Amparo à Pesquisa do Estado de Minas Gerais (FAPEMIG APQ‐04418‐22, RED‐00089‐23, and APQ‐04228‐24), Programa Nacional de Genômica e Saúde de Precisão ‐ Genomas SUS from the Brazilian Ministry of Health, and Instituto Nacional de Salud, Peru.

## CONFLICTS OF INTEREST

All authors declared no conflict of interest.

## AUTHOR CONTRIBUTIONS

M.M.S. and C.M. wrote the manuscript; F.R.‐S. designed the research; M.M.S., C.M., F.R.‐S., B.M., G.B.‐A., and L.F.‐C. performed the research; C.M., M.M.S., B.M., G.B.‐A., and L.F.‐C. analyzed the data; C.S., C.P., E.T.‐S., H.G., and T.D.O. contributed new reagents/analytical tools.

## Supporting information


Data S1

Data S2
